# Characterization of a new 5' splice site within the caprine arthritis encephalitis virus genome: evidence for a novel auxiliary protein

**DOI:** 10.1186/1742-4690-5-22

**Published:** 2008-02-29

**Authors:** Stephen Valas, Morgane Rolland, Cécile Perrin, Gérard Perrin, Robert Z Mamoun

**Affiliations:** 1 AFSSA-Niort, Laboratoire d'Etudes et de Recherches Caprines, 79012 Niort, France; 2 INSERM U577, Université Victor Segalen Bordeaux 2, 146 rue Léo Saignat, 33076 Bordeaux, France; 3 CNRS, UMR 5235 DIMNP UMII, UMI, Université Montpellier II, CC 107, place E. Bataillon, 34095 Montpellier cedex 5, France; 4 Department of Microbiology, University of Washington, Seattle, WA 98195-8070, USA

## Abstract

**Background:**

Lentiviral genomes encode multiple structural and regulatory proteins. Expression of the full complement of viral proteins is accomplished in part by alternative splicing of the genomic RNA. Caprine arthritis encephalitis virus (CAEV) and maedi-visna virus (MVV) are two highly related small-ruminant lentiviruses (SRLVs) that infect goats and sheep. Their genome seems to be less complex than those of primate lentiviruses since SRLVs encode only three auxiliary proteins, namely, Tat, Rev, and Vif, in addition to the products of *gag*, *pol*, and *env *genes common to all retroviruses. Here, we investigated the central part of the SRLV genome to identify new splice elements and their relevance in viral mRNA and protein expression.

**Results:**

We demonstrated the existence of a new 5' splice (SD) site located within the central part of CAEV genome, 17 nucleotides downstream from the SD site used for the *rev *mRNA synthesis, and perfectly conserved among SRLV strains. This new SD site was found to be functional in both transfected and infected cells, leading to the production of a transcript containing an open reading frame generated by the splice junction with the 3' splice site used for the *rev *mRNA synthesis. This open reading frame encodes two major protein isoforms of 18- and 17-kDa, named Rtm, in which the N-terminal domain shared by the Env precursor and Rev proteins is fused to the entire cytoplasmic tail of the transmembrane glycoprotein. Immunoprecipitations using monospecific antibodies provided evidence for the expression of the Rtm isoforms in infected cells. The Rtm protein interacts specifically with the cytoplasmic domain of the transmembrane glycoprotein *in vitro*, and its expression impairs the fusion activity of the Env protein.

**Conclusion:**

The characterization of a novel CAEV protein, named Rtm, which is produced by an additional multiply-spliced mRNA, indicated that the splicing pattern of CAEV genome is more complex than previously reported, generating greater protein diversity. The high conservation of the SD site used for the *rtm *mRNA synthesis among CAEV and MVV strains strongly suggests that the Rtm protein plays a role in SRLV propagation *in vivo*, likely by competing with Env protein functions.

## Background

Caprine arthritis encephalitis virus (CAEV) and ovine maedi-visna virus (MVV) are small-ruminant lentiviruses (SRLVs) that cause slow and persistent inflammatory diseases primarily in the joints, lungs, central nervous system, and mammary glands of sheep and goats [1]. *In vivo*, the predominant target cells of SRLV infection are of the monocyte/macrophage lineage [2,3]. Several lines of evidence suggest that SRLVs have evolved complex strategies to escape the host immune control. Virus exposure to the host immune response is limited because infected circulating monocytes do not express a threshold level of viral mRNA necessary to allow virus production [4], and only differentiated tissue macrophages are permissive to SRLV infection [4,5]. A large fraction of infectious particles accumulates in intracellular vesicles of SRLV-infected cells [3,4,6-9], sequestering virus from host defense mechanisms. Together, the nonproductive infection of circulating monocytes and the assembly of viral structural products in specific intracellular compartments, presumably promote efficient dissemination and persistence of virus into the host. However, cellular and viral factors involved in the control of SRLV expression are still largely unknown.

The genomic organization of SRLVs appears to be less complex than those of primate lentiviruses. In addition to the *gag*, *pol*, and *env *genes coding for the structural proteins and enzymes common to all retroviruses, SRLVs encode three auxiliary proteins, namely, Tat, Rev, and Vif. The SRLV Tat protein was initially described as a trans-activator protein which weakly enhances the transcription initiation from the viral promoter [10,11]. Recent studies demonstrating the incorporation of this protein into viral particles and its ability to mediate cell cycle arrest in the G2/M phase led to the conclusion that the SRLV Tat protein would better be considered as an accessory protein similar to the Vpr protein of the primate lentiviruses [12]. The Rev protein allows the cytoplasmic expression of the incompletely spliced SRLV mRNAs that encode the structural proteins [13,14]. Thus, Rev is required for virus gene expression and replication. The Vif protein acts at the late stage of virus formation and/or release [15], and is required for viral replication *in vivo *[16,17].

The expression of the various SRLV gene products is complex and temporally regulated [18-20]. The production of the full panel of the different spliced messages is achieved by alternative splicing using many splice sites, most of them being located in the *pol*/*env *intermediate region of the SRLV genome. The fine tuning of each viral mRNA level regulates the ratio of the different SRLV proteins. Initially, the multiply-spliced transcripts that encode the Tat and Rev regulatory proteins are predominant. Then, a Rev-mediated transition occurs to permit the cytoplasmic accumulation of singly-spliced and full-length RNA species encoding the viral structural and enzymatic proteins. In CAEV-infected cells, Vif and Env are expressed from different singly-spliced mRNAs, Tat and Rev are each encoded by at least two alternatively multiply-spliced mRNAs [18,21,22].

Here, we report the identification of a novel 5' splice (SD) site highly conserved in all SRLV genomes sequenced to date. The sequence of this SD site matches perfectly the canonical SD site. In CAEV-infected cells, the use of this SD site leads to an alternatively spliced mRNA that encodes two major protein isoforms of 18- and 17-kDa, designated Rtm. These proteins are expressed in infected cells and contain the N-terminal part of Env/Rev fused to the entire cytoplasmic domain of the transmembrane glycoprotein (TM). The Rtm proteins interact specifically with the cytoplasmic domain of TM *in vitro*, and modulate the fusion activity of viral envelope glycoproteins.

## Results

**Figure 1:**
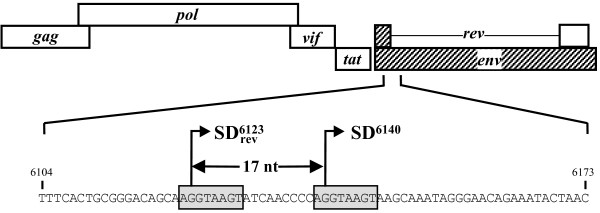
**Schematic representation of the SRLV ORFs**. The *env *sequence of the prototype CAEV (Cork) strain carrying the SD site used for the *rev *mRNA synthesis (SD^rev^) is enlarged. The nucleotide motifs corresponding to the canonical SD sequence are boxed, with splice points designated by bent arrows.

In an attempt to identify *cis*-acting viral element that would be the signature of new SRLV auxiliary proteins, we looked for sequences within the *pol*/*env *intermediate region of the CAEV Cork genome. We found, immediately downstream from the previously described SD site (SD^6123^) used for the *rev *mRNA synthesis [23,24], a sequence AGGTAAGT which was a perfect repeat of the SD^6123 ^sequence (Fig. 1). Interestingly, the SD^6123 ^site and this putative SD^6140 ^site were 17 nt distant from each other, and were consequently in different frames.

### The SD^6140 ^site is competent for splicing activity

**Figure 2:**
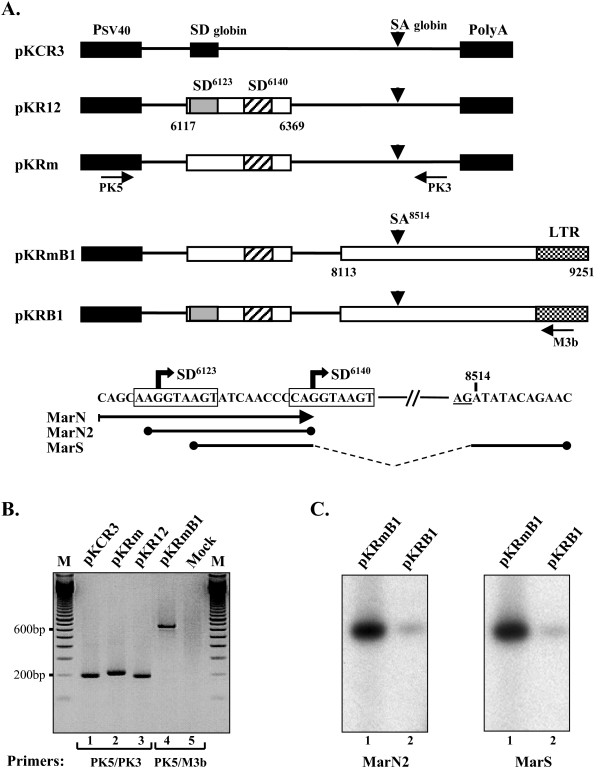
**Splicing activity assays of SD sites within the CAEV *env *gene**. *A*, Schematic representation of constructs used for splicing activity assays. Reporter constructs were based on the vector pKCR3 which contained the β-globin intron flanked by its splicing sequences inserted between the early promoter and poly-A site of SV40. CAEV sequences are included in open boxes. In all constructs, the β-globin SD site was replaced by CAEV sequences containing the SD^6123 ^(grey box) and SD^6140 ^(hatched box) sites. In plasmids pKRmB1 and pKRB1, the β-globin SA site was substituted by the 3' end viral genome containing the SA^8514 ^site. The positions of the primers used for PCR amplification of cDNA are indicated (horizontal arrows). The positions of probes MarN2 and MarS used in southern blot analysis are indicated. The MarN PCR primer used in experiment reported in Fig. 4 is indicated. *B*, RT-PCR analysis of RNAs extracted from transfected 293T cells. cDNAs were PCR amplified using primer pairs PK5 and PK3, or PK5 and M3b, as indicated. PCR products were resolved on an agarose gel and visualized by ethidium bromide staining. Lane M, DNA size markers. *C*, Southern blot analysis of transcripts from cells transfected with pKRmB1 and pKRB1 plasmids. PCR-amplified cDNAs were fractionated through a 2.5% agarose gel, blotted to nylon, and hybridized to probes MarN2 (left panel) and MarS (right panel).

To test whether the putative SD^6140 ^site corresponded to a *bona fide *SD site, we first analyzed the functionality of this element in a heterologous context (Fig. 2A). The original SD site of the rabbit β-globin intron in the parental pKCR3 plasmid was substituted by the viral sequence (nt 6117–6369) encompassing both the SD^6123 ^and SD^6140 ^sites (plasmid pKR12). In the plasmid pKRm, the upstream SD^6123 ^site was disrupted by a G^6124^→C mutation. For functional assay of the SD^6140 ^site, cytoplasmic RNAs were extracted from either pKRm or pKR12 transfected 293T cells and amplified by RT-PCR. As shown in Fig. 2B, the presence of the SD^6140 ^site alone induced efficient splicing of the rabbit β-globin intron (lane 2). As expected, the control pKR12 plasmid led to a shorter product (lane 3) originating from a splicing at the SD^6123 ^site. Similar result was obtained with plasmid pKRmB1, generated from the pKRm plasmid, in which the 3' splice (SA) site of the rabbit β-globin intron was substituted by 3' end of Cork proviral genome (nt 8813–9251) harboring the well described SA^8514 ^site used with the SD^6123 ^site to produce the *rev*-specific mRNAs (Fig. 2A). Indeed, a 660 nt signal corresponding to the expected SD^6140^/SA^8514 ^splicing product was detected from pKRmB1 transfected cells (Fig. 2B, lane 4).

Sequence analysis of the 660 nt PCR product confirmed the junction between the SD^6140 ^and SA^8514 ^sites (data not shown), demonstrating that the CAEV genome contains an additional SD site at position 6140, leading to a new splicing event within the Env coding region.

Analysis of RT-PCR fragments from cells transfected with plasmid pKR12 containing the native viral sequence revealed a spliced product shorter than that obtained with plasmid pKRm in which the SD^6123 ^was disrupted (Fig. 2B, compare lines 2 and 3), suggesting that no or few splicing occured at the SD^6140 ^site in the presence of the upstream SD^6123 ^site. To determine whether splicing activity at the SD^6140 ^site occurred or not in the presence of a functional SD^6123 ^site, Southern blot analysis was performed on RT-PCR products produced from cells transfected with either pKRB1 or pKRmB1 plasmids containing native or mutated SD^6123 ^site, respectively. Two radiolabeled probes were designed to specifically detect RNAs spliced at the SD^6140 ^site (Fig. 2A, bottom). The probe MarN2 was targeted against the sequence located between the SD^6123 ^and SD^6140 ^sites, while the probe MarS overlapped the splice junction between the SD^6140 ^and SA^8514 ^sites. As shown in Fig. 2C, the SD^6140 ^site promoted splicing of the SRLV *env *sequence even in the presence of the functional SD^6123 ^site (lanes 2). As expected, the splicing activity at the SD^6140 ^site greatly increased in the absence of the upstream competitive SD^6123 ^site (lanes 1). These results demonstrated the functionality of the SD^6140 ^site in the context of a wild-type viral sequence, and reinforced the potential complexity of the CAEV mRNA pool.

### Characterization of the *rtm *ORF

**Figure 3:**
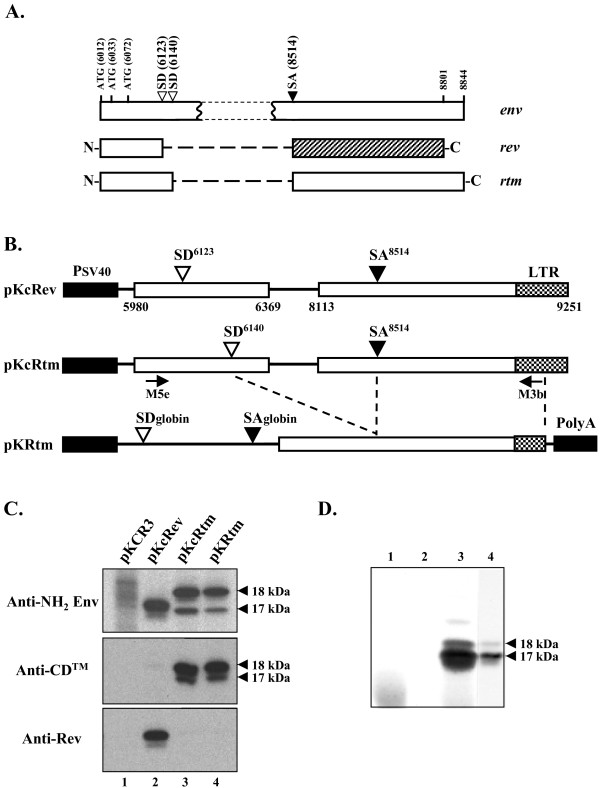
***rtm *ORF codes for two 18- and 17-kDa protein isoforms related to envelope precursor and TM proteins**. *A*, Relationships between domains shared by Env precursor, Rev and Rtm proteins. Splicing events within the Env coding region leading to *rev *and *rtm *ORFs are shown. Env precursor and Rev derived domains are represented by open and shaded boxes, respectively. *B*, Schematic representation of Rev and Rtm expression constructs. Plasmids pKcRev and pKcRtm are predicted to express singly-spliced mRNAs encoding the Rev and Rtm proteins, respectively. The pKRtm expression vector contains the *rtm *cDNA generated by RT-PCR from cells transfected with pKcRtm. The approximate positions of PCR primers are indicated (horizontal arrows). *C*, Coding capacity of the *rtm *ORF. Transfected 293T cells were radiolabeled 5 h with [^35^S]-methionine 48 h after transfection, and protein extracts were subjected to immunoprecipitation analysis using rabbit affinity-purified antibodies raised against either the first 38 amino acids of Env precursor (anti-NH_2 _Env), the 110-amino acid cytoplasmic domain of TM (anti-CD™), or the 98-amino acid carboxy terminus of Rev (anti-Rev). Immunoprecipitated proteins were resolved by electrophoresis through a SDS-15% polyacrylamide gel and visualized by autoradiography. *D*, Analysis of *in vitro *translation products of *rtm *cDNA. [^35^S]-methionine labeled polypeptides were synthesized in an *in vitro *coupled transcription-translation reaction with pGEM-1 (lanes 1 and 2) or *rtm *cDNA (lanes 3 and 4). Crude products (lanes 1 and 3) and proteins immunoprecipitated with affinity-purified anti-CD™ antibodies (lanes 2 and 4) were analyzed as described above.

The splice junction between the SD^6140 ^and SA^8514 ^sites predicted the existence of a novel ORF in which the N- and C-terminal parts of the Env precursor were merged together (Fig. 3A). Depending of the *env *initiation codon used (positions 6012, 6033, or 6072), the encoded proteins would contain either the first 43, 36 or 23 amino acids of the Env precursor fused to the entire 110-amino acid cytoplasmic domain of TM. These novel chimeric proteins, that we termed Rtm (for Rev-TM), would exhibit molecular masses of 17.8-kDa, 17-kDa and 15.5-kDa, respectively. Since the synthesis of the SRLV Rev protein is also initiated at the *env *initiation codon, the Env precursor, Rev and Rtm proteins would share a common N-terminal sequence. To test the coding ability of the *rtm *ORF, immunoprecipitation experiments were performed from 293T cells transfected with a Rtm expression plasmid. This expression vector (pKcRtm) was derived from the pKRmB1 plasmid in which the 5' end of the *rtm *ORF was reconstructed by inserting of the viral sequence containing the *env *initiation codon (Fig. 3B). Since *rev *and *rtm *ORFs predicted that both proteins had very similar sizes, the SD^6123 ^site was disrupted (G^6124^→C mutation) in the Rtm expression plasmid in order to improve the specificity of the detection of the protein. A Rev expression plasmid (pKcRev) was constructed as a control by using similar strategy, except that this plasmid contained a wild-type SD^6123 ^site and a mutated (G^6141^→C mutation) SD^6140 ^site (Fig. 3B). In order to identify the Env-derived domains within the Rtm protein, immunoprecipitations of ^35^S-labeled proteins expressed from transfected cells were performed by using three distinct antibodies developed by immunization of rabbits with GST fused proteins.

The specificities of these antibodies were as follows: i) anti-NH_2 _Env antibodies recognizing the 38 N-terminal amino acids of the Env precursor; ii) anti-CD™ antibodies recognizing the cytoplasmic domain of TM; iii) monospecific anti-Rev antibodies recognizing the 98 C-terminal amino acids of Rev. As shown in Fig. 3C, two major protein species of apparent molecular weights of 18- and 17-kDa expressed from cells transfected with the Rtm expression vector (lane 3) were immunoprecipitated with either anti-NH_2 _Env or anti-CD™ antibodies. A minor protein species with a size slightly smaller than 18-kDa was also immunoprecipitated with the anti-NH_2 _Env antibodies. None of these proteins were immunoprecipitated with monospecific anti-Rev antibodies. Two proteins exhibiting slightly different mobilities were immunoprecipitated from cells transfected with the Rev expression vector (lane 2) by using either anti-NH_2 _Env or anti-Rev antibodies, but not with anti-CD™ antibodies. No corresponding protein was immunoprecipitated from cells transfected with the empty parental plasmid pKCR3 (lane 1). These results demonstrated that the *rtm *ORF encoded two major protein isoforms carrying antigenic determinants derived from both the N- and C-termini of the Env precursor. As expected, these proteins did not share any antigenicity with the C-terminus of Rev. These two major protein isoforms of apparent molecular weights of 18- and 17-kDa corresponded likely to the expected proteins of 17.8- and 15.5-kDa, the minor band corresponding to the expected protein of 17-kDa. The fact that Rtm proteins were recognized by antibodies directed against both NH2 and COOH termini of the Env precursor indicated that they were not degradation products of the Env precursor.

Expression of two isoforms from cells transfected with the CAEV *rev *cDNA has been previously reported [13]. It has been suggested that they resulted from initiation at the first methionine codon (position 6012) and from leaky scanning and initiation at one of the two downstream in frame initiation codons (positions 6033 and 6072) within the *env *gene (Fig. 3A), leading to a protein of 15.3-kDa and to an isoform of either 14.5- or 13-kDa, respectively. Since *rev *and *rtm *ORFs shared the same 5' coding region, it was likely that the Rtm-related isoforms resulted from a similar leaky translational mechanism. Alternatively, they could originate from an alternative splicing removing part of the *rtm *ORF. To discriminate between these two hypotheses, immunoprecipitations were performed from cells transfected with the plasmid pKRtm carrying the fully spliced *rtm *cDNA (Fig. 3B), which was obtained by RT-PCR from cells transfected with plasmid pKcRtm. Two major proteins with similar mobilities and antigenic properties were produced from cells transfected with pKcRtm and pKRtm plasmids (Fig. 3C, compare lanes 3 and 4), indicating that these protein isoforms did not result from alternative splicing of the *rtm *transcript. To rule out any post-translational modifications or protein degradations, the *rtm *cDNA was used as a template in an *in vitro *transcription-translation reaction. As shown in Fig. 3D, analysis of the cell-free radiolabeled translated proteins also revealed the two Rtm proteins (lane 3), which were specifically immunoprecipitated by anti-CD™ antibodies (lane 4), whereas no product was detected in mock experiments (lanes 1 and 2). Interestingly, the fact that the *in vitro *18-kDa:17-kDa ratio was inversely related to that observed *in vivo *was in favor of a leaky scanning origin of the 17-kDa protein. Altogether, these results strongly suggested that the two isoforms of 18- and 17-kDa encoded by the *rtm *ORF resulted from translational initiation at different in frame start codons, as previously reported for Rev protein synthesis.

### Splicing activity at the SD^6140 ^site occurs in CAEV-infected cells, leading to the production of the *rtm *ORF

**Figure 4:**
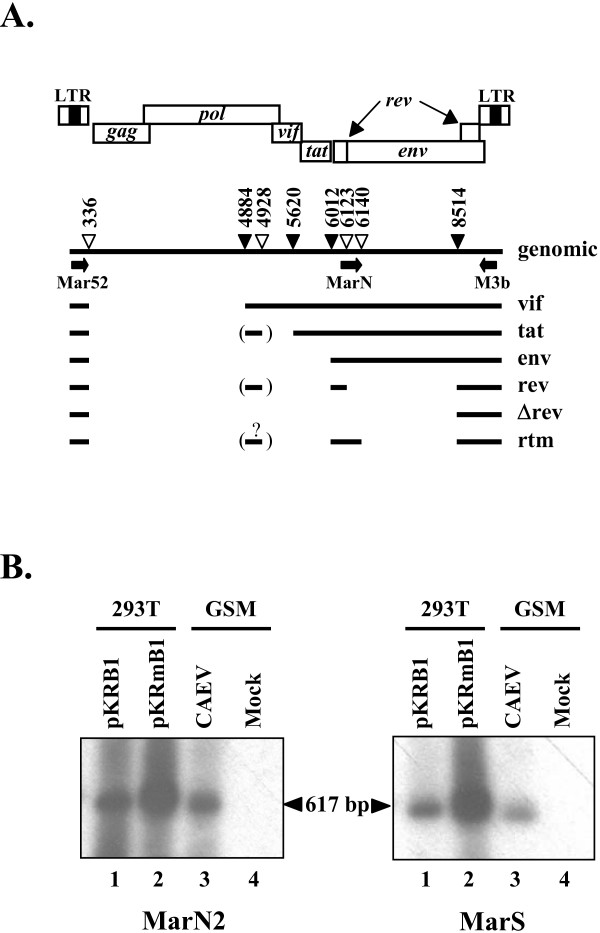
**Splicing junction between SD^6140 ^and SA^8514 ^sites occurs in CAEV-infected cells**. *A*, Proviral organization and splicing pattern of CAEV genome. The nucleotide numbers of SD sites (open triangles) and SA sites (solid triangles) are shown. All splice sites were identified by cDNA sequencing. Exons are represented by solid lines. Alternative exons which are present in only some of the mRNAs are shown in parenthesis. The putative structure of *rtm *transcript generated by splicing between SD^6140 ^and SA^8514 ^sites is shown. The arrows represent PCR primers used for cDNA amplification. *B*, Southern blot analysis of cDNAs from either transfected or infected cells. Cytoplasmic RNAs extracted from either 293T cells transfected with plasmids pKRB1 (lane 1) and pKRmB1 (lane 2) or CAEV-infected (lane 3) and non-infected (lane 4) GSM cells were submitted to RT/PCR. Primer pairs PK5/M3b and Mar52/M3b were used to amplify in a first-round PCR the cDNAs from transfected and infected cells, respectively. Primer pair MarN/M3b was used in the second-round PCR. PCR-amplified cDNA fragments were electrophoresed through an 2.5% agarose gel, blotted to nylon, and hybridized to either probe MarN2 (left panel) or probe MarS (right panel). Size of PCR-amplified fragments corresponding to the splice junction 6140–8514 is indicated.

**Figure 5:**
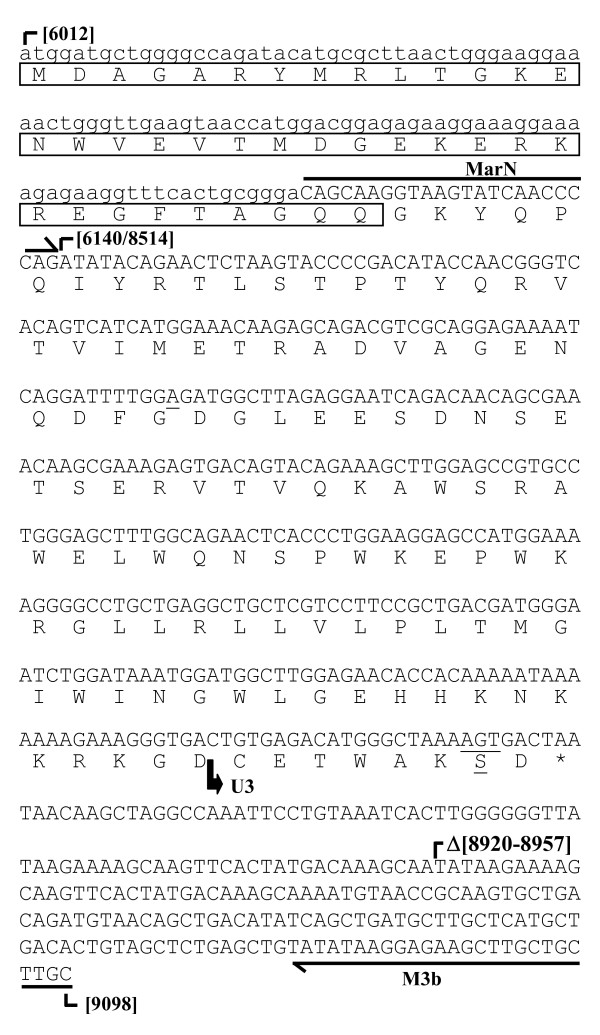
**Identification of a novel CAEV ORF**. The 617-bp cDNA amplified by nested-PCR from CAEV-infected GSM cells (see Fig. 4B) was cloned and sequenced. The region sequenced (uppercase letters) is bound by primers MarN and M3b (overlined) used in the second-round PCR. The region in lowercase letters is from the previously published CAEV nucleotide sequence. Numbers in brackets indicate the nucleotide positions of the CAEV genomic sequence (22). The predicted translation product (named Rtm) is shown below the sequence. The amino acids shared by the Rev and Rtm proteins are boxed. The nucleotide and amino acid substitutions of the cDNA compared to the previously published CAEV-Cork sequence are underlined. Deletion is represented by an open triangle. Stop codon is designated as asterisk.

To determine whether splicing activity at the SD^6140 ^site occurred in an infectious context, cDNAs from CAEV-infected GSM cells were amplified by RT/PCR, and then analyzed by Southern blot hybridization using probes MarN2 and MarS (Fig. 2A). The primers used in PCR were first Mar52 and M3b, located in the CAEV leader non-coding exon and the U3 region, respectively (Fig. 4A), and then MarN and M3b, allowing amplification of cDNAs corresponding to mRNAs generated by splicing at the SD^6140 ^site (Fig. 2A and 4A). As a control, cDNAs from 293T cells transfected with plasmids pKRB1 and pKRmB1 were obtained similarly, except that the forward Mar52 primer was substituted by the PK5 primer in the first round PCR (Fig. 2A). These controls led to a 617-bp amplified product specifically detected by both MarN2 and MarS probes (Fig. 4B, lanes 1 and 2), a size expected in view of the sequence of the plasmid used. The CAEV-infected GSM cells led to a slightly smaller product (designated as ~617-bp) revealed with both probes (Fig. 4B, lanes 3), whereas no product was detected from mock-infected GSM cells (Fig. 4B, lanes 4). The signals corresponding to *vif, tat*, and *env *singly-spliced transcripts were not observed, but such long fragments were not expected to be efficiently amplified using our experimental conditions. To find out the origin of the unexpected slight difference in size between products from infected and transfected cells, the ~617-bp cDNA amplified from CAEV-infected cells was cloned and sequenced (Fig. 5). Nucleotide sequence analysis revealed (i) the splice junction between the SD^6140 ^and SA^8514 ^sites, (ii) both synonymous (nt 8606) and nonsynonymous (nt 8838 to 8840) substitutions, and (iii) a 37 nt deletion (nt 8920 to 8957) within one copy of a duplicated 70-bp motif located in the U3 region downstream from the *rtm*, *rev *and *env *ORFs. All these features were confirmed by an independent experiment. This confirmed that the 37 nt deletion accounted for the size difference of RT/PCR products obtained from infected and transfected cells. Such deletion was previously found in some CAEV genomes (25). These results demonstrated that the splicing activity of the SD^6140 ^site also occurred in CAEV-infected cells, leading to the synthesis of the *rtm *ORF.

### Rtm is expressed in CAEV-infected cells

**Figure 6:**
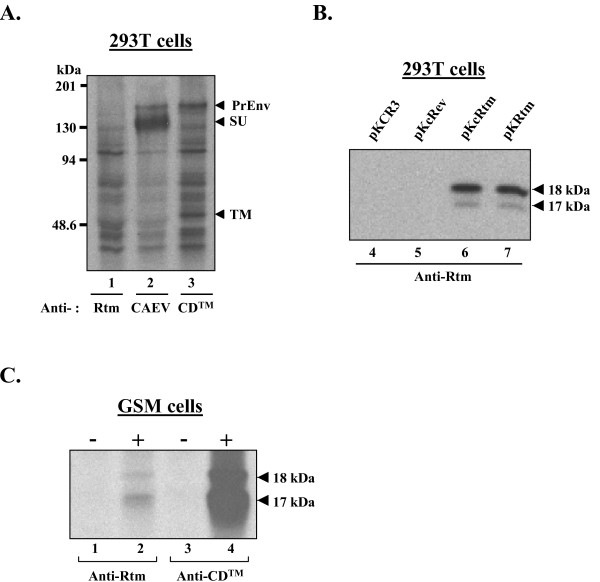
**Detection of Rtm expression in transfected 293T cells and infected GSM cells using a Rtm-specific peptide antiserum**. *A and B*, Specificities of rabbit anti-Rtm peptide antibodies. 293T cells were transfected with Env (pKEnv) plasmid (A), or with either parental (pKCR3), Rev (pKcRev), or Rtm (pKcRtm and pKRtm) plasmids, as indicated (B). Cells were radiolabeled 5 h with [^35^S]-methionine 48 h after transfection. Lysates were subjected to immunoprecipitation and fractionated on an SDS-10% polyacrylamide gel. Immunoprecipitations were performed using either anti-CD™ antibodies, a serum from CAEV-infected goat (anti-CAEV), or anti-Rtm peptide antibodies (anti-Rtm). *C*, Immunoprecipitation of the Rtm protein from infected cells. GSM cells were either mock infected (-) or infected with CAEV-Cork strain (+). When cytopathic effects appeared in infected cell culture, cells were radiolabeled 5 h with [^35^S]-methionine, and lysates were immunoprecipitated with either anti-Rtm or anti-CD™ antibodies, as indicated. Immunoprecipitated proteins were resolved on a SDS-15% polyacrylamide gel.

To investigate whether Rtm protein was expressed in CAEV-infected cells, we needed to rule out numerous drawbacks including common antigenic determinants shared by the Rtm, Env and Rev proteins, and similar molecular weights of Rtm and Rev proteins. Considering that a Rtm-specific epitope might be encoded by the nucleotide sequence overlapping the splice junction specific to the *rtm *mRNA, we generated a rabbit antiserum against the synthetic peptide (KYQPQIYRT) corresponding to the translated product of this specific Rtm coding region. First of all, the specificity of these anti-Rtm antibodies was tested by immunoprecipitation of [^35^S]-radiolabeled proteins produced from transfected cells. As shown in Fig. 6A, the anti-Rtm antibodies immunoprecipitated neither the Env precursor nor the mature SU and TM glycoproteins produced from cells transfected with an Env expression vector (lane 1), while these Env products were recovered by immunoprecipitation using either CAEV-infected goat serum or anti-CD™ rabbit serum (lanes 2 and 3, respectively). Similarly, no protein was immunoprecipitated by the anti-Rtm antibodies from mock-transfected cells or cells transfected with the Rev expression vector (Fig. 6B, lanes 4 and 5, respectively). In contrast, the anti-Rtm antibodies recognized both Rtm isoforms produced from cells transfected with the Rtm expression vectors (Fig. 6B, lanes 6 and 7), demonstrating that this rabbit antiserum was specific to the Rtm protein.

Next, we determined whether Rtm protein was expressed in CAEV-infected cells. A lysate from GSM cells infected by the CAEV-Cork strain was immunoprecipitated with either anti-Rtm or anti-CD™ antibodies. The two proteins of 18- and 17-kDa were clearly detected by both types of antibodies in infected cells whereas no product was detected in uninfected cells (Fig. 6C, compare lanes 2 and 4 with lanes 1 and 3). The signal using the anti-Rtm antibodies was faint compared with that obtained with the anti-CD™ antibodies, indicating probably a low peptide-antibody affinity. We concluded that the Rtm protein was expressed in CAEV-infected GSM cells.

To know whether the Rtm protein was expressed in infected animals we looked for humoral immune response against it. Considering that most antibodies that would recognize the Rtm protein might in fact result from an immune response against the Rev and/or Env proteins, we tested for the presence of antibodies recognizing the KYQPQIYRT Rtm-specific epitope. For this purpose, fifty milliliters of pooled sera from three seropositive goats experimentally infected with the CAEV-Cork strain were loaded onto a resin matrix covalently bound with the Rtm peptide. After extensive washing, bound antibodies were eluted and tested by ELISA using either the Rtm peptide or the GST-CD™ protein as antigens and by Western blot using the GST-CD™ protein. None of these assays provided positive results. We concluded that if the Rtm was expressed in infected animals the KYQPQIYRT epitope was not enough immunogenic to give rise to the production of antibodies or that these antibodies did not recognize the synthetic peptide.

### Rtm protein interacts with the cytoplasmic domain of TM

**Figure 7:**
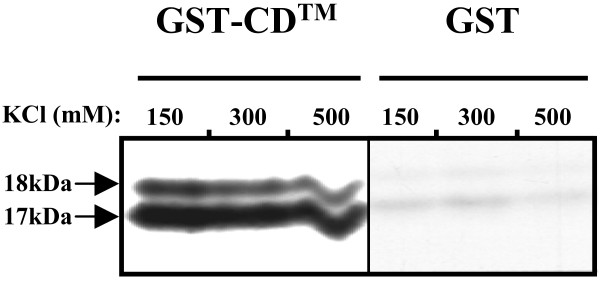
**Rtm binds to the cytoplasmic domain of Env protein**. Purified recombinant GST-CD™ or GST alone were incubated with *in vitro*-translated Rtm protein labeled with [^35^S]methionine. The beads were washed six times in the presence of different ionic strengths of KCl, as indicated, and the bound proteins were subjected to 15% SDS-PAGE analysis and autoradiography.

Considering that the major part of the Rtm sequence corresponded to the cytoplasmic domain of TM and that the homologue domain of HIV TM was reported to self-assemble as an oligomer [26], we looked for an interaction between the Rtm and the cytoplasmic domain of TM. In this attempt, a GST pull-down assay was performed to identify potential interaction of Rtm with Env protein. *In vitro*-translated, radiolabeled Rtm protein was incubated with either a GST fusion protein containing the entire cytoplasmic domain of TM (GST-CD™) or with GST alone coupled to glutathione-Sepharose beads. Equal amounts of protein were used in all binding experiments, as verified by SDS-PAGE and Coomassie blue staining (data not shown). After extensive washing of the bead-bound complexes in different stringent conditions, the bound proteins were analysed by SDS-PAGE and autoradiography. As shown in Fig. 7, the Rtm protein interacted with the GST-CD™, and this interaction was resistant to high ionic strength washes. In contrast, no significant interaction was observed in association with the GST protein alone.

These results clearly indicated that Rtm protein strongly and specifically interacts with the cytoplasmic domain of TM in vitro.

### The SD^6140 ^site is strictly conserved throughout the SRLV phylum

**Figure 8:**
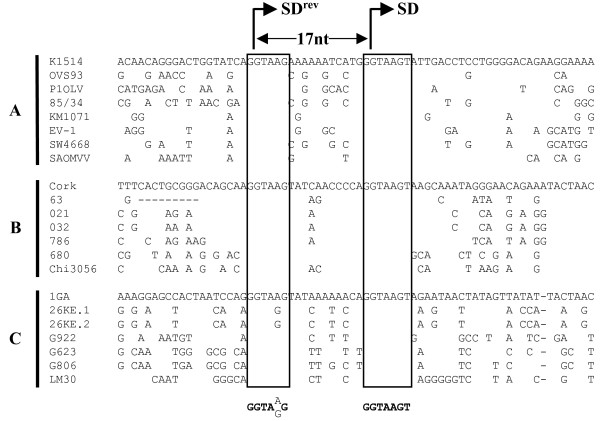
**Genetic homologies of the internal exon sequence carrying the SD^rev ^and SD^rtm ^sites among SRLV strains**. All available sequences belonging to the three phylogenetic groups (A, B and C) are aligned. Dashes indicate deletions. A consensus sequence is presented. The conserved SD motifs are boxed.

To assess the biological importance of the SD^6140 ^site and of the Rtm protein for SRLVs, we assumed that it should be conserved in all SRLV genomes, as the *rev *SD^6123 ^site is. To look for the conservation of the SD^6140 ^site among SRLV strains, previously described env sequences representative of highly divergent phylogenetic clusters were aligned (Fig. 8). This alignment confirmed that the 5' region of the SRLV *env *gene was extremely variable, except two quasi perfect repeat sequences (GGTAAG) corresponding to the SD^6123 ^and SD^6140 ^sites of Cork genome. Remarkably, the 17 nt distance between the two SD sites was also perfectly conserved among all SRLV sequences. Moreover, the downstream SD site was even better conserved than the SD site used for the *rev *mRNA synthesis. The high conservation of all these genetic features (sequence, frame, and nt distance) within a highly variable region strongly suggested that the Rtm protein, like the rev protein, is very important for SRLVs.

### Rtm protein affects the fusion activity of Env protein

Given the importance of the cytoplasmic domain of TM in several Env protein functions such as Env sorting and membrane fusion activity and the specific interaction between this domain and Rtm protein in our *in vitro *binding assay, we looked for the effects of Rtm expression on the Env fusion activity. For this purpose, 293T cells were transfected with pKEnv plasmid or cotransfected with pKEnv and pKRtm plasmids, then fusion activity was assessed by coculture with GSM target cells. In all transfections, the plasmid amounts were normalized by addition of empty plasmid pKCR3 in order to have the same copy number of SV40 promotor. As shown in Table 1, the expression of Rtm reduced by 79% the syncytium number with respect to that obtained with Env protein expressed alone.

**Table 1:** Effects of Rtm on the fusion activity of Env protein.

Plasmid^a^	No. of syncytia/well (293T/GSM cocultivation)^b^	% of syncytia^c^
pKCR3	0	0
pKEnv	954 ± 105	100
pKEnv + pKRtm	200 ± 46	21

^*a *^The plasmid amounts were normalized by addition of empty plasmid pKCR3 in order to have the same copy number of SV40 promotor in all transfections.

^*b *^Numbers of syncytia in 30 randomly selected fields per well. Averages of three wells are shown.

^*c *^Number of syncytia divided by the number obtained with the positive control (pKEnv + pKCR3 plasmids) × 100.

## Discussion

A novel CAEV protein, designated Rtm, was characterized on the basis of its immunological cross-reactivity with antibodies directed against either the N-terminus of the Env precursor or the C-terminus of TM, and by cDNA sequence analysis which established that *rtm *ORF results from a novel splicing junction linking the 5' and 3' part ends of the *env *gene. Immunoprecipitation experiments using monospecific antibodies raised against the peptide encoded by the sequence straddling this splice junction confirmed the existence of the *rtm *ORF. Like Rev, the Rtm expression was initiated at the *env *ORF start codons and led to the production of two major protein isoforms of 18- and 17-kDa. Previous studies together with this work indicated that both Rev and Rtm protein doublets may be attributed to a leaky scanning at the first AUG initiation codon and initiation at one of the two downstream in frame *env *initiation codons [13,23,24]. The ratio between the 18- and 17-kDa isoforms depends on the context and/or the level of the Rtm expression, the 18-kDa being the major product in transfected 293T cells whereas the 17-kDa is the major product in infected GSM cells and in the *in vitro *transcription/translation reaction.

The *rtm *ORF was generated by splicing between the SD^6140 ^site identified in this study and the well described SA^8514 ^site. Since the *rev *ORF is produced by splicing between the SD^6123 ^and SA^8514 ^sites, the Rev and Rtm proteins are expressed from distinct but structurally closely related multiply-spliced mRNAs. The SD^6140 ^site matched perfectly the canonical SD sequence and was highly conserved among all SRLV genomes. Moreover, the distance between the SD^6123 ^and SD^6140 ^sites is also strictly conserved among SRLV sequences, despite the variability of the surrounding region. Altogether, these results strongly suggested that the Rtm protein is an additional auxiliary factor required for successful SRLV propagation *in vivo*. The Rtm protein is a chimeric protein in which the N-terminal part of the envelope precursor is joined to the complete cytoplasmic domain of TM. An expression of this domain in a viral envelope-free context has been also reported for MVV in a previous study showing that a 16.5-kDa protein corresponding to the C-terminal domain of TM is produced in an *in vitro *transcription/translation reaction from the *rev *transcript by leaky scanning and initiation at a downstream initiation codon [23]. For SRLVs, equine infectious anemia virus (EIAV), bovine and simian (SIV) immunodeficiency viruses, the 5' terminal part of the *env *gene also constitutes the first coding exon of Rev. Thus, Rtm could exhibit some functions of its related proteins, Rev and/or TM. Several studies demonstrated that motifs involved in CAEV Rev function are exclusively localized in the C-terminal domain of the protein [13,27]. Moreover, a truncated Rev protein devoid of its N-terminal domain is still functional [21], although this domain has been shown to be responsible for optimal binding to the Rev responsive element [28]. Our attempts to identify a Rev activity in Rtm were unsuccessful (data not shown). Altogether, these results strongly indicate that Rtm does not harbor any Rev activity. More likely, the biological functions of Rtm would be harbored by its C-terminus which corresponds precisely to the entire cytoplasmic domain of TM.

The cytoplasmic domain of the TM protein is considerably longer for lentiviruses than for other retroviruses [29], and has been reported to be involved in multiple crucial steps of virus infection, such as particle assembly, infectivity, replication, and pathogenesis. For HIV and SIV, the cytoplasmic domain of TM modulates Env fusogenicity, expression on the cell surface, and incorporation into viral particles as well as the biochemical and immunologic properties of the Env ectodomain [30-39]. The underlying mechanisms responsible for the regulation of Env protein function involve association of the cytoplasmic domain of TM with cellular and viral components [40-50]. These interactions require canonical tyrosine-based and di-leucine motifs as well as small domains called lentivirus lytic peptide. Since most of these signals were found in the cytoplasmic domain of CAEV TM (Fig. 5), it was likely that similar mechanisms have been developed by SRLVs to regulate the Env protein function. Consequently, expression of the cytoplasmic domain of TM in a viral glycoprotein-free context like Rtm could potentially interfere with Env protein function. We showed here that the Env-mediated syncytium induction is dramatically reduced in the presence of Rtm protein. Using a GST pull-down assay, we also found that Rtm protein interacts specifically with the cytoplasmic domain of TM. Altogether, these findings suggest that the Rtm protein acts in the infectious cycle of SRLV by modulating the Env protein function through direct interaction with the cytoplasmic domain of TM and/or through competitive interaction with cellular factors.

Assuming that Rtm acts as a down regulator of Env protein function, it is likely that Rtm is not expressed during the late phase of the viral cycle when Env proteins must be highly expressed, correctly sorted and incorporated in the virions. This is sustained by the fact that despite Rtm interacts strongly with the cytoplasmic domain of the TM, all our attempts to detect Rtm in virions failed (data not shown). Moreover, Rtm is translated from a multiply-spliced transcript, arguing in favor of its expression early during the viral replication cycle since previous studies have revealed a temporal shift from multiply-spliced to mono- and unspliced SRLV transcripts [20,22]. Since the Rev protein is absolutely required for the expression of the viral structural proteins, we can hypothesize that expressions of Rev and Rtm proteins are temporally dissociated from each other. This assumption can accommodate with the fact that SRLV expression is restricted in monocytes, and upregulated during maturation of monocytes into macrophages [4,5]. In latently infected monocytes, virus replication is blocked at a post-transcriptional stage [2,4], which could result from the downregulation effects of Rtm. Differentiation of monocytes into macrophages is also required for EIAV expression [51]. Interestingly, a very similar mRNA encoding a Rtm-like protein has been described in an acutely infected horse [52]. This hybrid protein, designated Ttm, contains the domain encoded by the first coding exon of Tat fused to the complete cytoplasmic tail of TM. Similarly to the Rev-derived sequence in the Rtm protein, the Tat-derived sequence in the Ttm protein does not contain functional domains of the native protein. Thus, the role of the cytoplasmic domain of TM expressed in these two similar hybrid proteins would be interesting to examine in the context of the high variation of viral expression of these two macrophage-tropic lentiviruses in their hosts.

## Conclusion

In the present study we have identified a new competent SD site at position 6140 in the CAEV genome that presents two remarkable features. First, despite its location within a highly variable region, the sequence of this SD site is perfectly conserved among CAEV and MVV strains, the only other conserved stretch of nucleotides in this region corresponding to the SD site used for the *rev *mRNA synthesis. Second, the 17 nt distance between the two SD sites is also strictly conserved in the genome of all SRLV isolates. Splicing at the SD^6140 ^site was demonstrated in both transfected and CAEV-infected cells, leading to the production of an ORF encoding two major protein isoforms of 18- and 17-kDa, named Rtm. These proteins are generated by differential translation initiation, contain the N-terminal part of the Env precursor fused to the complete 110-amino acid cytoplasmic domain of TM, and are expressed in CAEV-infected cells. The exceptional degree of conservation of the SD^6140 ^site sequence among CAEV and MVV isolates and the fact that *rtm *and *rev *transcripts were structurally closely related strongly suggest that the Rtm protein is a fourth important auxiliary factor of SRLVs. We showed that this protein interacts specifically with the cytoplasmic domain of TM and dramatically affects the fusion activity of Env protein. Altogether, these findings support a model of regulation of SRLV expression by a new viral factor, which can accommodate confirmed observations regarding SRLV infection *in vivo*. In this aspect, the functional activity of the cytoplasmic domain of the TM expressed in a viral glycoprotein-free context should be considered to better understand the parameters of SRLV propagation *in vivo*.

## Methods

### Cells and sera

Primary foetal goat synovial membrane (GSM) cells infected with CAEV-Cork strain were maintained in culture in Eagle's minimal essential medium supplemented with 1% glutamine, 200 U/ml penicillin, 200 μg/ml streptomycin, 5 μg/ml fungizone, and 10% foetal bovine serum (FBS). The human 293T cell line was propagated in Dulbecco's modified Eagle's medium (DMEM) supplemented with 1% glutamine, 25 μg/ml gentamycin, and 5% FBS. CAEV-specific sera were collected from goats experimentally infected with CAEV-Cork strain. Rabbit polyclonal antisera against the cytoplasmic domain of TM (anti-CD™), the N-terminus of Env precursor (anti-NH_2 _Env), and the C-terminus of Rev (anti-Rev) were generated by using GST fusion proteins as immunogens. Purified fusion proteins were used in association with Freund adjuvant to immunize New Zealand White rabbits. Rabbit antisera were affinity purified on GST fusion protein/affi-gel 10 columns according to the manufacturer's instructions (BIO-RAD).

A synthetic peptide derived from the predicted Rtm open reading frame (ORF) product (amino acids 39 to 47) was designed for the production of monospecific anti-Rtm antibodies. This Rtm-specific peptide (KYQPQIYRT) was synthesized using a 9-fluorenylmethyloxycarbonyl chemical strategy (Sigma-Genosys) and rabbit peptide-specific antiserum was generated by immunization with the peptide cross-linked to the keyhole limpet hemocyanin carrier.

### Plasmid constructions

All eukaryotic expression plasmids were derived from the pKCR3 vector [53] in which the rabbit β-globin intron 2 flanked by its splice sites was inserted between the early promoter and polyA signal site of the simian virus 40 (SV40). The viral sequences were derived from both the 9-kbp and 0.5-kbp *Hin*dIII clones of CAEV-Cork strain [22,54]. The nucleotides (nt) are numbered according to the Cork sequence [22]. To generate constructs used for splicing activity assays, a fragment (nt 6117 to 6674) spanning the well-described SD^6123 ^site and the putative SD^6140 ^site was PCR-amplified from the 9-kbp *Hin*dIII clone, and subcloned into the *Sma*I site of pGEM-1 plasmid. Disruption of the SD^6123 ^site (G to C substitution at nt 6124) was achieved with primer 5'-*TCTAAAGGATCCCC*CAGCAAGCTAAGTATCAACCCCAG-3' (non CAEV sequences are shown in italic) by using the CLONTECH Transformer site-directed mutagenesis kit (mutated nt are underlined in the nucleotide sequences). A 261-bp fragment containing either the wild-type or mutated viral sequence was double digested with *Bam*HI (in the multiple cloning site [MCS] of pGEM-1) and *Hin*cII (position 6369 in Cork sequence), and cloned in place of the original β-globin SD site in the pKCR3 plasmid, to generate pKR12 and pKRm plasmids, respectively. The plasmids pKRB1 and pKRmB1 were generated by inserting the *Apa*I-*Bam*HI fragment of the 0.5-kbp *Hin*dIII clone (nt 8113 to 9251) into the *Apa*I-*Bgl*II-digested pKR12 and pKRm plasmids, respectively.

To generate eukaryotic expression plasmids coding for Env, Rev and Rtm proteins, the *Spe*I-*Bam*HI fragment of the 9-kbp *Hin*dIII clone, containing the *env *initiation codon (nt 6012) and both the SD^6123 ^and SD^6140 ^sites, was cloned into the *Xba*I/*Bam*HI-digested pGEM-1 plasmid. To facilitate cloning, a *Bam*HI site was introduced by site-directed mutagenesis at position 5979, upstream the *env *initiation codon, using the primer 5'-TGCAAATAAATGGATCCAACAAGTAGCAAAAGT-3' (nt 5968 to 6000). Mutagenic primers 5'-GGGACAGCAAGCTAAGTATCAA-3' (nt 6113 to 6134) and 5'-TATCAACCCCAGCTAAGTAAGCAA-3' (nt 6129 to 6152) were used to disrupt the SD^6123 ^and SD^6140 ^sites, respectively. The resulting 231-bp *Bam*HI-*Eco*RV fragments (nt 5980 to 6211) containing either the mutated SD^6123 ^site or the mutated SD^6140 ^site were cloned into the similarly-digested pKRmB1 to produce pKcRtm and pKcRev plasmids, respectively. The pKEnv plasmid was generated by cloning the *Eco*RI-*Bst*XI fragment (nt 6348 to 8368) of the 9-kbp *Hin*dIII clone into the similarly-digested pKcRev plasmid. The Plasmids encoding the Rtm protein from the cDNA were generated as follows. Two PCR primers were used to amplify cDNA from 293T cells transfected with pKcRtm. Forward primer M5e (5'-GGAATTCATGGATGCTGGGGCCAGATAC-3'; nt 6012 to 6032) and reverse primer M3b (5'-CGGGATCCGCAAGCAGCAAGCTTCTCCTTATATA-3'; nt 9098 to 9073) contained *Eco*RI and *Bam*HI sites at their 5' end, respectively. The *Eco*RI-*Bam*HI digested PCR product was cloned into pKCR3 digested with *Eco*RI and *Bgl*II to produce plasmid pKRtm. The same digested PCR product was cloned under the T7 promoter control between the *Eco*RI/*Bam*HI sites of pGEM-1, generating the pGRtm plasmid used as template in cell-free transcription/translation reaction. All clones were verified by sequencing.

### RNA isolation and RT-PCR

RNAs were extracted from either transfected 293T cells or infected GSM cells with the RNA Now extraction kit (Biogentex) according to the manufacturer's protocol. Five micrograms of Dnase-treated RNAs were used for reverse transcription in a final volume of 20 μl containing 200 U of Moloney murine leukemia virus reverse transcriptase (Promega), 1 mM of each deoxynucleoside triphosphate (dNTPs), 1 mM DTT, 500 ng of oligo(dT)_12–18 _and 26 U of RNase inhibitor (Amersham). Reverse transcription (RT) was carried out for 15 min at 37°C and then for 45 min at 42°C. PCR amplifications were carried out in a final volume of 50 μl of 1× PCR buffer (Perkin-Elmer), with 200 μM (each) dNTPs, 2 mM MgCl_2_, 100 ng of each primer, 0.5 U AmpliTaq DNA polymerase (Perkin-Elmer), and 10 μl of cDNA, using the following conditions: 3 min denaturation at 94°C, followed by 35 amplification cycles of 40 sec at 94°C, 50 sec at 53°C, 50 sec at 72°C, followed by a final 4 min extension step at 72°C. Nested-PCR reactions were performed under similar conditions using one-tenth of the RT-PCR products as templates. PCR products were separated by 2.5% agarose electrophoresis and visualized by ethidium bromide staining. After blotting onto a nylon membrane (Hybond-N^+^, Amersham), the membrane was prehybridized for 3 h at 46°C in 5× SSC (1× SSC is 0.15 M NaCl plus 0.15 M sodium citrate), 0.5% sodium dodecyl sulfate (SDS), 100 μg/ml salmon sperm DNA. The prehybridization solution was then replaced by the hybridization solution containing the ^32^P-labeled oligonucleotide probe. After overnight incubation at 46°C, the membrane was washed four times with 2× SSC – 0.1% SDS at room temperature for 15 min and then once with 1× SSC – 0.1% SDS at 46°C for 15 min before being exposed to X-ray film (Kodak) at -70°C. The oligonucleotide used to detect messages spliced at the SD^6140 ^site was MarN2 (5'-AGGTAAGTATCAACCCCAG-3'; nt 6122 to 6140). The oligonucleotide used to detect Rtm-specific spliced message was MarS (5'-AGTATCAACCCCAGATATACAGAAC-3'; nt 6127 to 6140 and nt 8514 to 8524), which overlapped the splice junction between the SD^6123 ^and SA^8514 ^sites.

Two primer pairs were alternatively used in a first round PCR reaction to amplify cDNAs from transfected cells: PK5 (5'-TAGTGAGGAGGCTTTTTTGGAG-3'; forward primer) and PK3 (5'-GAAGATCTCAGTGGTATTTGTGAGCCA-3'; reverse primer), both located in pKCR3 sequence, or PK5 and M3b. The primer pair used in a first round PCR reaction to amplify cDNAs from infected GSM cells were Mar52 (5'-TAATCTGTGCAATACCAGAGCGGCT-3'; nt 131 to 155; forward primer) and M3b. Primer pair MarN (5'-CAGCAAGGTAAGTATCAACCCCAG-3'; nt 6117 to 6140; forward primer) and M3b was used in a second round PCR reaction.

### Construction of GST fusion proteins

Plasmids pGST-NH_2 _Env, pGST-Rev, and pGST-CD™ encode the gluthatione S-transferase (GST) fused C-terminally to the N-terminus of Env precursor (amino acids 1 to 38), the C-terminus of Rev (amino acids 36 to 133), and the cytoplasmic domain of TM (amino acids 203 to 312), respectively. To generate pGST-NH_2 _Env, a PCR product was amplified from pGRtm by using primers M5e and 5'-CGCGGATCCTTGCTGTCCCGCAGTGAAACCT-3', digested with *Eco*RI-*Bam*HI, and then cloned into the *Eco*RI/*Bgl*II sites of pGEX-A (Pharmacia). To generate the GST-Rev fusion protein, cDNA from 293T cells transfected with pKRB1 was used as template to amplify by PCR the Rev C-terminus coding region by using primers PK5 and M3x (5'-TGTCTAGAGCAAGCAGCAAGCTTCTCCTTATATA-3'; nt 9098 to 9073). The PCR product was digested with *Eco*RI-*Xba*I and then cloned into the corresponding sites of pGEM-1. Then, a *Bam*HI/*Hin*cII fragment encoding the C-terminal domain of Rev was excised and subcloned into the *Bam*HI/*Sma*I sites of pGEX-3X (Pharmacia). Similar cloning strategy was applied to cDNA from 293T cells transfected with pKRmB1 to generate pGST-CD™. GST fusion proteins were produced in *Escherichia coli *strain DH5α and purified by using Glutathione-Sepharose 4B beads (Pharmacia Biotech) by standard procedures [55].

### Metabolic labeling and immunoprecipitation

The 293T cells were transfected by the calcium phosphate coprecipitation technique [56]. At 48 h post-transfection, 293T cells were incubated for 30 min in methionine/cysteine-free DMEM supplemented with 5% dialyzed FBS and then metabolically labeled for 5 h with 100 μCi of [^35^S] methionine/cysteine mixture (Promix; Amersham) per ml. Cells were then lysed in 1× RIPA buffer (10 mM Tris-HCl [pH 7.8], 1 mM Na_2_HPO_4_, 1 mM EDTA, 0.5% NP-40, 0.5% sodium deoxycholate, 0.2 mM PMSF), and cell lysates were cleared by centrifugation at 15,000 × *g *for 15 min at 4°C. Immunoprecipitation reactions were performed by incubating cell lysates with protein A-Sepharose beads and either 300 μl of affinity-purified rabbit antibodies or 5 μl of serum from CAEV-Cork infected goat preadsorbed on normal 293T cell lysate, overnight at 4°C with rocking. The bead-antibody-protein complexes were washed five times with RIPA buffer, boiled in sample loading buffer (60 mM Tris-HCl [pH 6.8], 1% SDS, 1% β-mercaptoethanol, 10% glycerol, 0.01% bromophenol blue), and recovered proteins were subjected to SDS-15% polyacrylamide gel electrophoresis (SDS-PAGE) analysis. The gel was fixed, soaked in Amplify (Amersham) for 30 min, dried and autoradiographied.

### Cell fusion activity

The 293T cells were plated (2 × 10^5 ^cells per well) in triplicate in six-well plates in complete DMEM the day prior transfection. The cells were transfected with 1.5 μg of pKEnv plasmid or cotransfected with 1.5 μg of pKEnv plasmid and 1.5 μg of pKRtm plasmid. The copy number of the SV40 promotor was normalized in each transfection by adding appropriate amounts of empty plasmid (pKCR3). On the next day, primary GSM cells (2.5 × 10^5 ^cells per well) were added to transfected cells. One day post-coculture, cells were fixed and stained to visualize nuclei. Fusion activity was assessed by counting syncytia in 30 randomly selected fields at 100× magnification of each well.

### *In vitro *transcription and translation

One microgram of linearized plasmids pGRtm or pGEM-1 were transcribed and translated in a coupled rabbit reticulocyte lysate system (Promega) according to the manufacturer's instructions with T7 RNA polymerase and 0.8 μCi/μl of [^35^S] methionine/cysteine mixture (Amersham) in a final volume of 50 μl. The translated products were analyzed on SDS-PAGE and detected by autoradiography. Immunoprecipitations of translation products with affinity purified anti-CD™ antibodies were carried out as described above.

### GST pull-down assays

Equal amounts, as judged by Coomassie blue staining, of the different GST fusion proteins complexed to glutathione-Sepharose beads were incubated with 10 μl of [^35^S]-labeled Rtm protein produced from *in vitro *transcription/translation reaction, in binding buffer (10 mM Tris [pH 7.4], 10% glycerol, 0.2 mM EDTA, 0.5 mM DTT, 0.25% Triton X-100) supplemented with 80 mM KCl and 100 μg/ml of bovine serum albumin in a final volume of 300 μl overnight at 4°C with constant agitation. The beads were washed six times in binding buffer containing different concentrations of KCl (150 mM, 300 mM, or 500 mM), boiled in sample loading buffer, and the proteins were subjected to 15% SDS-PAGE analysis and autoradiography as described above.

## Competing interests

The author(s) declare that they have no competing interests.

## Authors' contributions

SV performed most of the laboratory work, MR contributed to the Western blot analyses, and CP contributed to the preparation of serum samples. GP and RZM conceived the strategies and designed the experiments. SV and RZM wrote the manuscript. All authors read and approved the final manuscript.
